# Co-occurrence of health risk behaviors and depression in the Brazilian population: analysis of a population-based survey (Vigitel, 2023)

**DOI:** 10.1590/1980-549720260028

**Published:** 2026-07-27

**Authors:** Bruna Carolina Rafael Barbosa, Laudicéia Ferreira Fróis, Mariana Cassemira Aparecida Vidigal, Melissa Ionara Ribeiro Sabbagh, Diovana Raspante de Oliveira Souza, Aline Silva de Aguiar, Ísis Eloah Machado, Adriana Lúcia Meireles

**Affiliations:** I Universidade Federal de Ouro Preto, Graduate Program in Health and Nutrition – Ouro Preto (MG), Brazil.

**Keywords:** Ultra-processed food, Sedentary behavior, Tobacco use disorder, Binge drinking, Risk factor, Depression

## Abstract

**Objective:**

To analyze the association between the co-occurrence of health risk behaviors and the presence of depression in Brazilians.

**Methods:**

This was a cross-sectional study using data from the 2023 population-based telephone survey Vigitel. Depression was assessed by self-reported medical diagnosis. The co-occurrence of health risk behaviors was measured based on the sum of five behaviors: consumption of ultra-processed foods (UPF), physical inactivity, sedentary behavior, smoking, and binge drinking. This variable was subsequently categorized as “none,” “one,” “two,” or “three or more” behaviors. Descriptive analyses, Pearson’s χ^2^ test, and univariate and multiple logistic regression adjusted for sex, age, marital status, and level of education were carried out.

**Results:**

A total of 21,690 individuals participated in the survey, of whom 12.3% had depression, and approximately 79.8% reported at least one health risk behavior. Consumption of UPF (OR 1.35; 95%CI 1.08–1.68), physical inactivity (OR 1.29; 95%CI 1.04–1.60), and smoking (OR 1.76; 95%CI 1.36–2.27) were associated with depression. Individuals with two health risk behaviors had 1.36 times higher odds of depression (OR 1.36; 95%CI 1.08–1.73), while those with three or more had twice the odds (OR 2.01; 95%CI 1.49–2.71), compared to those with none.

**Conclusion:**

Individuals with the co-occurrence of two or more health risk behaviors are more likely to have depression. These findings highlight the importance of integrated strategies for mental health promotion and the prevention of unhealthy behaviors in the Brazilian population.

## INTRODUCTION

Depression is an important public health issue and one of the main causes of disability and economic burden^1,2^. The World Health Organization (WHO) estimates that more than 300 million people worldwide suffer from depression^3^, affecting approximately 3.8% of the population, including 5% of adults — 4% men and 6% women — and 5.7% of individuals over 60 years of age^4^. It is estimated that depression contributed to approximately 56 million (95% uncertainty interval: 39.3–76.5) of disability-adjusted life years (DALYs) globally in 2021^5^, and may become the second largest cause of global disease burden by 2030^6^.

In Brazil, according to data from the National Survey of Health (*Pesquisa Nacional de Saúde* – PNS), there was an increase in the prevalence of self-reported diagnosis of depression among adults (≥18 years), from 7.6% in 2013 to 10.2% in 2019, which corresponds to about 16.3 million individuals diagnosed with depression in the country^7^. These findings indicate a significant increase in the number of depression diagnoses over the years and emphasize the severity of the issue.

The etiology of depression is multifactorial, resulting from a complex interaction between biological, social, psychological, and behavioral factors^8^. Among the behaviors, health risk behaviors stand out, which include inadequate eating, physical inactivity, sedentary behavior (SB), smoking, and binge drinking^9,10,11^. In addition, according to the literature, these behaviors, especially smoking and binge drinking, are important risk factors for mortality among the population with depression^12^.

Researchers have shown that the simultaneous presence of multiple health risk behaviors is associated with an increased risk of mental disorders, including anxiety symptoms, depression, and the concomitant occurrence of both^10,13^. Although many studies evaluate these behaviors in isolation^11,14,15,16,17^, there is a growing interest in approaches that consider co-occurrence. This is due to the fact that the adoption of a health risk behavior is often linked to the presence of another, with its manifestation being often accompanied by co-occurrence than alone, which can potentiate the negative effects^9,10,13^.

Understanding the association between health risk behaviors and depression is essential for the development of public health strategies and interventions that promote mental health and encourage healthy habits. These actions can contribute to reducing chronic noncommunicable diseases (NCDs) among the population, improving quality of life and physical and mental well-being. Thus, the objective of this study was to analyze the association between the co-occurrence of health risk behaviors and depression in Brazilians.

## METHODS

This is a cross-sectional study with data from the Surveillance System for Risk and Protective Factors for Chronic Diseases by Telephone Survey (*Sistema de Vigilância de Fatores de Risco e Doenças Crônicas por Inquérito Telefônico* – Vigitel) conducted in 2023. Vigitel is a national population-based survey that, since 2006, monitors the population (≥18 years) living in the capitals of the Brazilian states and the Federal District. Through telephone interviews, the survey monitors the frequency and distribution of the main risk and protective factors for NCDs in Brazil^18^.

The Vigitel sampling process occurred in two stages. In the first one, landlines and mobile phone lines were drawn per city. In the second stage, an adult (≥18 years) was selected for the interview, drawn among the residents of the household. The sampling included 800 interviews per city, 400 of which were conducted by landlines and 400 by mobile phones. This methodology ensured estimates with a 95% confidence interval (95%CI) and a margin of error of up to four percentage points for the frequency of risk and protective factors in the adult population of each locality. Detailed information on the Vigitel sampling process can be found in the published report^18^.

### Study variables

#### Outcome variable — depression

The presence of depression was defined by the question: “*Have any doctor ever informed you that you have depression?*”, being categorized as “yes” or “no.”

#### Explanatory variable — co-occurrence of health risk behaviors

The co-occurrence of health risk behaviors was evaluated based on the sum of five behaviors: consumption of ultra-processed foods (UPF), physical inactivity, sedentary behavior (SB), smoking, and binge drinking.

The consumption of UPF was analyzed based on a question regarding the consumption of 13 food subgroups on the day prior to the interview: “*Now I’m going to list some foods and I’d like you to tell me if you ate any of them yesterday (since the moment you woke up until you went to bed): sweetened drinks (soft drinks, ready-made juices, powdered refreshments, chocolate drinks), industrialized snacks (packaged snacks, cookies, candies and savory pastries, cupcakes), processed desserts (chocolates, ice cream, gelatins, flans), processed meats (sausages, mortadella, ham), industrialized breads (sandwich bread, hot dog bun, burger bun), condiments (mayonnaise, ketchup, mustard), margarines, and readymade meals (noodles, instant soups, frozen dishes*)”. The UPF subgroups were selected among those most consumed in Brazil, according to the Consumer Expenditure Survey (*Pesquisa de Orçamentos Familiares* – POF) of 2008–2009^19^. For analysis purposes, a score was calculated based on the positive responses for the consumption of each of the UFP subgroups, ranging from 0 to 13 points. Based on the final score, UPF consumption was considered high when equal to or greater than five subgroups, being categorized as “no” (<5 subgroups) or “yes” (≥5 subgroups)^20^.

Physical inactivity was evaluated based on the classification of participants as physically inactive. Those who, in the last three months, did not practice any physical activity (PA) in their leisure time, did not perform intense physical efforts at work, did not commute on foot or by bicycle to work or to school/courses, and did not perform heavy household chores were considered physically inactive. This indicator was developed based on Vigitel’s questions evaluating the four PA domains: leisure, commute, occupational, and domestic^18^.

SB was evaluated by screen time, considering the questions: “*On average, how many hours a day do you usually watch television*?” and “*On average, how many hours of your leisure time (excluding work) are occupied by using a computer, tablet, or mobile phone?*”. Participants were classified “without SB” or “with SB,” the latter category being attributed to those who reported three or more hours a day in SB^18^.

Smoking was measured by the question: “*Currently, do you smoke?*”. Individuals who responded positively, regardless of the number of cigarettes, frequency, or duration of the smoking habit, were considered smokers.

To evaluate binge drinking, the following questions were used, aimed at men and women, respectively: “*In the last 30 days, did you have 5 or more drinks of alcoholic beverages on a single occasion*?” and “*In the last 30 days, did you have 4 or more drinks of alcoholic beverages on a single occasion?*”. A drink of alcoholic beverage was considered equivalent to a can of beer, a glass of wine, or a dose of *cachaça*, whiskey or any other distilled alcoholic beverage. Binge drinking was defined as the intake of five or more drinks, in the case of men, or four or more drinks, in the case of women, on a single occasion compared to the last 30 days prior to the date of the survey^18^.

Based on the responses to the five behaviors, a variable of co-occurrence of health risk behaviors was created, whose score ranged from zero to five. For analysis purposes, this variable was categorized into: “none,” “one,” “two,” and “three or more” health risk behaviors.

### Covariates

The following sociodemographic and anthropometric characteristics of the individuals were evaluated: sex (men/women); age (18–24; 25–34; 35–44; 45–54; 55–64; ≥65 years); level of education (0–8; 9–11; ≥12 years of formal study); marital status (with or without partner/spouse); and overweight, defined as body mass index (BMI) ≥25 kg/m^2^, calculated according to self-reported weight and height^18^.

### Statistical analysis

Descriptive analyses were performed by frequency distribution (%) and respective 95%CI. Pearson’s χ^2^ test was used to evaluate the relationship between covariates and the presence of depression.

The association between each health risk behavior, individually, and the presence of depression was evaluated by univariate logistic regression. Subsequently, multiple logistic regression analysis was carried out between the explanatory variable and the outcome, with crude estimates adjusted for the covariates evaluated in this study. Odds ratio (OR) was the association measure, considering 95%CI. Adjustment variables were selected based on evidence from the literature on association with depression and health risk behaviors^21,22,23^.

For data analysis, the Stata software version 18.0 was used in the survey module, which considers the effects of complex sampling and allows extrapolating the results to the Brazilian population. A 5% significance level was adopted in all statistical analyses.

### Ethical aspects

The Vigitel project was approved by the National Ethics Committee on Research Involving Human Beings of the Ministry of Health (CAAE: 65610017.1.0000.0008). Vigitel databases are of public access and can be consulted on the official website of the Department of Epidemiological Analysis and Surveillance of Chronic Noncommunicable Diseases (*Departamento de Análise Epidemiológica e Vigilância de Doenças Não Transmissíveis* – DAENT): https://svs.aids.gov.br/download/Vigitel/.

#### Data availability statement

Vigitel data are available for public access at the following URL: https://svs.aids.gov.br/daent/cgdnt/vigitel/

## RESULTS

A total of 21,690 individuals were evaluated, most of whom were women, aged between 25 and 54 years, with level of education between 9 and 11 years, without a partner, and overweight. The medical diagnosis of depression was reported by 12.3%. Except for the age variable, all other covariates were related to depression (Table 1).

**Table 1:** Sociodemographic and anthropometric characteristics according to the presence of depression among Brazilian adults and older adults (n=21,690). Vigitel, 2023.

Variables	Frequency (%, 95%CI)	Depression (%, 95%CI)		p-value
		Absence	Presence	
		87.7 (86.8–88.5)	12.3 (11.5–13.2)	
Sex				
Men	46.0 (44.5–47.6)	92.9 (91.8–93.9)	7.1 (6.1–8.2)	**<0.001**
Women	54.0 (52.4–55.5)	83.2 (81.9–84.5)	16.8 (15.5–18.2)	
Age (years)				
18 to 24	12.9 (11.8–14.0)	89.2 (86.1–91.6)	10.8 (8.4–13.9)	0.152
25 to 34	24.9 (23.4–26.4)	88.9 (86.7–90.7)	11.1 (9.3–13.3)	
35 to 44	18.3 (17.2–19.4)	86.5 (84.3–88.4)	13.5 (11.6–15.7)	
45 to 54	18.3 (17.2–19.5)	88.2 (86.2–90.0)	11.8 (10.0–13.8)	
55 to 64	13.9 (12.9–14.8)	86.6 (84.5–88.5)	13.4 (11.6–15.6)	
≥65	11.7 (11.1–12.5)	85.7 (84.0–87.4)	14.3 (12.6–16.1)	
Level of education (years)				
0 to 8	32.9 (31.6–34.3)	86.0 (84.3–87.5)	14.0 (12.5–15.7)	**0.016**
9 to 11	41.3 (39.8–42.8)	89.0 (87.6–90.2)	11.0 (9.9–12.4)	
≥12	25.8 (24.4–27.2)	87.8 (85.9–89.4)	12.2 (10.6–14.1)	
Marital status				
Have a partner	46.5 (45.0–48.0)	89.1 (88.0–90.2)	10.9 (9.7–12.1)	**0.001**
Have no partner	53.5 (52.0–55.0)	86.3 (84.9–87.5)	13.7 (12.5–15.1)	
Overweight				
No	38.6 (37.2–40.1)	89.0 (87.7–90.2)	11.0 (9.8–12.3)	**0.015**
Yes	61.4 (59.9–62.8)	86.8 (85.6–88.0)	13.2 (12.0–14.4)	

Note: Depression: assessed based on self-report.

BMI: Body mass index ≥25 kg/m^2^ (overweight).

p-value obtained by Pearson’s χ^2^ test.

Variables that presented statistical significance are in bold.

Among the evaluated behaviors, SB was the most prevalent, corresponding to 67.04% of the participants (Table 2).

We observed that the consumption of UPF, physical inactivity, and smoking are associated with the presence of depression. Participants with high UPF consumption have higher odds of depression (OR 1.35; 95%CI 1.08–1.68; p=0.008) compared to those with UPF consumption <5 subgroups. Physically inactive individuals had higher odds of depression (OR 1.29; 95%CI 1.04–1.60; p=0.021) when compared to active individuals. In addition, smokers were more likely to experience depression (OR 1.76; 95%CI 1.36–2.27; p<0.001), compared to nonsmokers (Table 2).

**Table 2:** Association between health risk behaviors and depression among Brazilian adults and older adults (n=21,690). Vigitel, 2023.

Health risk behaviors	Prevalence (%)	Depression			
		Crude analysis OR (95%CI)	p-value	Adjusted analysis OR (95%CI)	p-value
Consumption of ultra-processed foods					
No	82.25	1		1	
Yes	17.75	1.12 (0.91–1.38)	0.284	1.35 (1.08–1.68)	**0.008**
Physical inactivity					
No	86.12	1		1	
Yes	13.88	1.29 (1.04–1.60)	**0.021**	1.30 (1.05–1.62)	**0.018**
Sedentary behavior					
No	32.96	1		1	
Yes	67.04	1.11 (0.94–1.31)	0.220	1.11 (0.93–1.32)	0.233
Smoking					
No	90.73	1		1	
Yes	9.27	1.76 (1.36–2.27)	**<0.001**	2.09 (1.60–2.73)	**<0.001**
Binge drinking					
No	79.24	1		1	
Yes	20.76	0.99 (0.80–1.23)	0.921	1.18 (0.95–1.47)	0.131

Note: Values in bold indicate statistical significance (p<0.05).

Multiple logistic regression adjusted for sex, age, marital status, and level of education.

Ultra-processed food consumption: categorized as “no” (<5 subgroups) and “yes” (≥5 subgroups).

Physical inactivity: classified as “no” or “yes.” Participants who did not perform any physical activity in the last three months were considered physically inactive in any of the four evaluated domains: leisure, commute, occupational, and domestic.

Sedentary behavior: categorized as “no” for individuals without sedentary behavior and “yes” for those who reported having three or more hours of sedentary behavior a day.

Smoking: categorized as “no” and “yes.” Individuals who reported to smoke were considered smokers, regardless of the number of cigarettes, frequency, or duration of the habit.

Binge drinking: classified as “no” or “yes,” being considered “yes” when having five or more drinks, in the case of men, or four or more drinks, in the case of women, on a single occasion.

According to the multiple regression analysis between the co-occurrence of health risk behaviors and depression, we verified a dose-response gradient (p<0.001), indicating that the odds of presenting depression increases the higher the number of health risk behaviors. The presence of only one behavior was not associated with depression. Participants with two behaviors were 1.36 times more likely to present with depression (OR 1.36; 95%CI 1.08–1.73; p<0.010), while those with three or more behaviors had higher odds (OR 2.01; 95%CI 1.49–2.71; p<0.001) compared to those with no health risk behavior (Table 3).

**Table 3:** Association between the co-occurrence of health risk behaviors and depression among Brazilian adults and older adults (n=21,690). Vigitel, 2023.

Number of health risk behaviors	Prevalence (%, 95%CI)	Depression		p-value
		Crude analysis (OR)	Adjusted analysis (OR)	
None	20.17 (19.04–21.36)	–	–	<0.001^*^
One	43.21 (41.73–44.71)	0.98 (0.80–1.20)	1.02 (0.83–1.26)	0.839
Two	26.26 (24.96–27.60)	1.21 (0.97–1.53)	1.36 (1.08–1.73)	**0.010**
Three or more	10.35 (9.38–11.41)	1.50 (1.12–2.00)	2.01 (1.49–2.71)	**<0.001**

Note: *p-trend referring to the linear trend test.

Values in bold indicate statistical significance (p<0.05).

Health risk behaviors: consumption of ultra-processed foods, physical inactivity, sedentary behavior, smoking, and binge drinking. Multiple logistic regression adjusted for sex, age, marital status, and level of education.

## DISCUSSION

According to the study results, which included more than 21 thousand Brazilian adults and older adults, we verified a prevalence of 12.3% of medical diagnosis of depression and 36.61% of the participants presented two or more health risk behaviors. The individual analysis of behaviors indicated that UPF consumption, physical inactivity, and smoking were associated with depression. Furthermore, a statistically significant association was observed between the co-occurrence of health risk behaviors and the presence of depression, evidencing that the increase in the number of behaviors was related to higher odds of depressive diagnosis.

According to data from *The Burden of Mental Disorders in the Region of the Americas* report, depression is the most disabling mental disorder in the Americas, accounting for about 7.8% of years lived with disability (YLDs)^6^. In Brazil, the scenario becomes particularly worrisome, as national population-based surveys, such as Vigitel and PNS, show a growing and continuous trend in depression rates over the last decades^7,19^.

The increase in the prevalence of depression is particularly worrisome when associated with the lifestyle of the population, especially those related to health risk behaviors. There is evidence of a bidirectional relationship between depression and inadequate food intake, SB, and other behaviors, configuring a vicious cycle that compromises both physical and mental health^9,24,25^.

In the present study, the consumption of UPF was associated with the presence of depression, corroborating the findings of Hecht et al.^26^. These authors observed that individuals with higher UPF intake were more likely to report mild depression, a higher number of days with mental suffering, and anxiety symptoms, as well as a lower probability of reporting absence of mental health impairment.

The association between frequent intake of UPF and increased levels of depressive symptoms^27^ can be explained by the adverse effects of these foods on metabolic health and neuropsychological functioning^28^. Researchers indicate that the consumption of UPF can trigger systemic inflammation through the activation of pro-inflammatory pathways and increased permeability of the intestinal barrier, favoring the development of intestinal dysbiosis. This inflammatory process can compromise neurotransmission, especially in the serotonergic and dopaminergic systems, which play a key role in mood regulation^29,30^.

In the present study, physical inactivity was associated with higher odds of depression, in line with evidence according to which inappropriate movement behaviors are related to mental disorders^15,16^. There is evidence that these behaviors influence mental health through changes in brain function and mood regulation^31,32^. In addition, different levels of PA intensities may have protective effects on depression, acting through biological and psychosocial mechanisms^16,33^.

Physical inactivity is associated with a reduction in the release of neurotransmitters essential for mood and well-being regulation such as dopamine, serotonin, and endorphin^31,34^. Moreover, the absence of PA compromises important neurobiological processes such as neurogenesis and brain plasticity^33,34^. Physically inactive individuals are more prone to the accumulation of psychological stress, as PA plays a key role in regulating stress response systems, such as the hypothalamic-pituitary-adrenal (HPA) axis^31,35^, neuroendocrine system responsible for the proper physiological response to stressful situations^33^.

Several mechanisms have been proposed to explain the association between SB and depression. One hypothesis is that high levels of SB may have direct deleterious effects on mood, regardless of other factors, possibly through changes in inflammatory processes, which constitute one of the main biological characteristics of major depression^16^. SB is also associated with increased systemic inflammation, evidenced by high levels of inflammatory markers, such as C-reactive protein and interleukin-6, which may affect the functioning of the central nervous system, favoring neurochemical and structural changes that increase the risk of developing depression^16,35,36^.

Another hypothesis suggests that screen-based SB, such as prolonged use of computers, may contribute to the reduction of face-to-face social interactions, which, in turn, would be associated with increased risk of developing mental disorders^16,36^. Finally, long periods spent on SB may result in a reduction in the practice of PA, which leads to a decrease in the release of neurotransmitters associated with positive mood regulation, such as endorphins — thus contributing to depressive symptoms^36^.

Besides movement behaviors, smoking was also associated with the presence of depression in this study, corroborating Völker et al.^37^. In a study with over 170 thousand participants, the authors observed that individuals who were former smokers had higher odds of depression throughout their lives and at the present time, in addition to an association between higher cigarette consumption and greater severity of depressive symptoms^37^.

Tobacco use has been identified as an important risk behavior for depression, with effects possibly mediated by neurobiological and inflammatory mechanisms. There is evidence that smokers have higher levels of depression compared to nonsmokers^27,37^, suggesting that smoking habit may negatively affect brain neurochemistry and favoring the development of depressive symptoms^38^. Nicotine, the main psychoactive substance of tobacco, stimulates the release of neurotransmitters in the brain reward system, generating pleasurable effects^39^. Neve theless, its chronic use may lead to the deregulation of these neurotransmitters, contributing to the development of depression^40^. In addition, smoking is associated with a systemic inflammatory state, characterized by the increase of pro-inflammatory cytokines, which may compromise neurogenesis and affect brain regions involved in mood regulation^41^.

In this study, the co-occurrence of health risk behaviors was associated with depression. We observed that the higher the number of risk behaviors, the higher the odds of depression, corroborating the findings of Barbosa et al.^9^ in a cross-sectional study conducted with 1,353 young adults in which the authors evaluated the co-occurrence of obesogenic behaviors and the presence of anxiety and depression symptoms. Furthermore, in a longitudinal study, Kekäläinen et al.^42^ found accumulated associations between health risk behaviors and mental well-being over 30 years, from early adulthood to later stages. The presence of multiple behaviors, such as smoking, binge drinking, and physical inactivity, was associated with worse mental well-being.

According to studies, health risk behaviors often tend to occur jointly, and that their co-occurrence may have a synergistic effect, enhancing the influence of one behavior over the others^9,13,21^. In addition, authors of epidemiological studies show that this combination is associated with mental health outcomes^9,42^.

Different mechanisms may explain the association between health risk behaviors and mental disorders. Changes in eating habits, for example, can reflect emotional eating or lack of motivation to maintain healthy dietary patterns. In this context, individuals with greater perception of stress and depressive symptoms tend to increase the consumption of UPF as a way of dealing with emotions^9^. These foods have been associated with increased depressive symptoms, possibly due to their negative effects on metabolic and neuropsychological health. Likewise, the literature suggests that smoking can influence brain neurochemistry and inflammation patterns, contributing to depression^27^.

Although our results are of great relevance, it is essential to consider some limitations. The cross-sectional design of the study does not allow us identify temporality or establish cause-effect relationship. It is worth highlighting the possibility of reverse causality, particularly with regard to depression and risk behaviors, including smoking, inadequate eating habits, and physical inactivity. The composition of the sample, restricted to individuals with access to landlines or mobile phones, may compromise the representativeness of the data in relation to the general population, especially in more vulnerable populations. The self-reported nature of the information also presents methodological challenges, exposing the data to potential biases of memory and interpretation. To mitigate these limitations, weighting factors were applied, seeking to adjust the sociodemographic composition of the sample, increasing both the representativeness and analytical robustness of the study. Finally, it should be noted that the combined assessment of risk behaviors limits the investigation of possible interactions and more complex patterns, unlike approaches such as cluster analysis.

Even in the face of these limitations, this study presents relevant contributions in elucidating the association between health risk behaviors and depression in the Brazilian population. The applied methodology enabled us not only to identify the associations, but also to highlight the interdependence between various behaviors harmful to health and their impact on depression. This holistic approach emphasizes the importance of health strategies that simultaneously integrate actions aimed at mental health and the promotion of healthy life habits.

The originality of this article lies in the combined analysis of multiple health risk behaviors and their interaction with mental health, offering a more comprehensive and detailed perspective on two important public health issues: NCDs and mental health. Our findings reinforce the need for comprehensive public policies, capable of articulating different strategies and promoting an integrated view on health determinants. Interventions that stimulate the regular practice of PA and encourage adequate and healthy dietary patterns, combined with the simultaneous reduction of health risk behaviors, can play a key role in the prevention and control of NCDs and mental disorders. The incorporation of these actions into the daily life of the population represents an opportunity to promote health in a broader and more sustainable way.
