# Supplementary material for: Co-occurrence of health risk behaviors and depression in the Brazilian population: analysis of a population-based survey (Vigitel, 2023)
Source: Rev Bras Epidemiol. 2026 Jul 27;29:e260028. doi: 10.1590/1980-549720260028 (PMC13405554; doi:10.1590/1980-549720260028)
Supplement: Translation

# Translation: Coocorrência de comportamentos de risco à saúde e depressão na população brasileira: análise de um inquérito de base populacional (Vigitel, 2023)

**DOI:** 10.1590/1980-549720260028.2

**Authors:** Bruna Carolina Rafael Barbosa, Laudicéia Ferreira Fróis, Mariana Cassemira Aparecida Vidigal, Melissa Ionara Ribeiro Sabbagh, Diovana Raspante de Oliveira Souza, Aline Silva de Aguiar, Ísis Eloah Machado, Adriana Lúcia Meireles

**Affiliations:** I Universidade Federal de Ouro Preto, Programa de Pós-Graduação em Saúde e Nutrição – Ouro Preto (MG), Brasil.

**Keywords:** Alimentos ultraprocessados, Comportamento sedentário, Tabagismo, Consumo excessivo de bebidas alcoólicas, Fator de risco, Depressão

## Abstract

**Objetivo:**

Analisar a associação entre a coocorrência de comportamentos de risco à saúde e a presença de depressão em brasileiros.

**Métodos:**

Estudo transversal com dados do inquérito de base populacional Sistema de Vigilância de Fatores de Risco e Doenças Crônicas por Inquérito Telefônico (Vigitel) 2023. A depressão foi avaliada pelo autorrelato de diagnóstico médico. A coocorrência de comportamentos de risco à saúde foi mensurada com base na soma de cinco comportamentos: consumo de alimentos ultraprocessados (AUP), inatividade física, comportamento sedentário, tabagismo e consumo abusivo de álcool. Posteriormente, essa variável foi categorizada em: “nenhum”, “um”, “dois” ou “três ou mais” comportamentos. Foram realizadas análises descritivas, teste de qui-quadrado de Pearson e regressão logística univariada e múltipla ajustada por sexo, idade, estado civil e escolaridade.

**Resultados:**

Participaram 21.690 indivíduos, sendo que 12,3% apresentaram depressão, e aproximadamente 79,8% relataram algum comportamento de risco à saúde. Observou-se que o consumo de AUP (RC 1,35; IC95% 1,08–1,68), a inatividade física (RC 1,29; IC95% 1,04–1,60) e o tabagismo (RC 1,76; IC95% 1,36–2,27) estão associados à presença de depressão. Indivíduos com dois comportamentos de risco à saúde tinham 1,36 vezes mais chance (RC 1,36; IC95% 1,08–1,73) de apresentarem depressão, enquanto aqueles com três ou mais tiveram 2,01 vezes mais chance (RC 2,01; IC95% 1,49–2,71), em comparação com aqueles que não apresentavam nenhum comportamento.

**Conclusão:**

Indivíduos que apresentam coocorrência de dois ou mais comportamentos de risco à saúde têm mais chance de depressão. Esses achados reforçam a importância de estratégias integradas de promoção da saúde mental e prevenção de comportamentos não saudáveis na população brasileira.

## INTRODUÇÃO

A depressão é um importante problema de saúde pública e uma das principais causas de incapacidade e ônus econômico^1,2^. A Organização Mundial da Saúde (OMS) estima que mais de 300 milhões de pessoas no mundo sofrem de depressão^3^, afetando aproximadamente 3,8% da população, incluindo 5% dos adultos — sendo 4% dos homens e 6% das mulheres — e 5,7% dos indivíduos com mais de 60 anos^4^. Estima-se que a depressão tenha contribuído com aproximadamente 56 milhões (intervalo de incerteza de 95%: 39,3-76,5) de anos de vida ajustados por incapacidade (*Disability-Adjusted Life Years* – DALYs) globalmente em 2021^5^, podendo se tornar a segunda maior causa de carga global de doenças até 2030^6^.

No Brasil, dados da Pesquisa Nacional de Saúde (PNS) evidenciam um aumento na prevalência de diagnóstico autorreferido de depressão entre adultos (≥18 anos), passando de 7,6% em 2013 para 10,2% em 2019, o que corresponde a cerca de 16,3 milhões de indivíduos com depressão diagnosticada no país^7^. Esses achados indicam um aumento significativo no número de diagnósticos de depressão ao longo dos anos e reforçam a gravidade do problema.

A etiologia da depressão é multifatorial, resultante de uma complexa interação entre fatores biológicos, sociais, psicológicos e comportamentais^8^. Entre os comportamentos, destacam-se os de risco à saúde, que incluem alimentação inadequada, inatividade física, comportamento sedentário (CS), tabagismo e consumo abusivo de álcool^9,10,11^. Além disso, a literatura aponta que esses comportamentos, especialmente o tabagismo e o consumo abusivo de álcool, são importantes fatores de risco para a mortalidade entre a população com depressão^12^.

Estudos têm evidenciado que a presença simultânea de múltiplos comportamentos de risco à saúde está associada a um risco aumentado de transtornos mentais, incluindo sintomas de ansiedade, depressão e a ocorrência concomitante de ambos^10,13^. Embora muitos estudos avaliem esses comportamentos de forma isolada^11,14,15,16,17^, há um interesse crescente em abordagens que considerem a coocorrência. Isso se justifica pelo fato de que a adoção de um comportamento de risco à saúde está frequentemente interligada à presença de outro, tornando mais comum sua manifestação conjunta do que sua ocorrência isolada, o que pode potencializar os efeitos negativos^9,10,13^.

Compreender a associação entre os comportamentos de risco à saúde e a depressão é essencial para a elaboração de estratégias em saúde pública e intervenções que promovam a saúde mental e incentivem hábitos saudáveis. Essas ações podem contribuir para a redução de Doenças Crônicas não Transmissíveis (DCNT) entre a população, melhorando a qualidade de vida e o bem-estar físico e mental. Assim, o objetivo deste estudo foi analisar a associação entre a coocorrência de comportamentos de risco à saúde e depressão em brasileiros.

## MÉTODOS

Estudo transversal com dados do Sistema de Vigilância de Fatores de Risco e Doenças Crônicas por Inquérito Telefônico (Vigitel), realizado em 2023. O Vigitel é um inquérito nacional de base populacional que, desde 2006, monitora a população (≥ 18 anos) residente nas capitais dos 26 estados brasileiros e no Distrito Federal. Por meio de entrevistas telefônicas, o inquérito acompanha a frequência e a distribuição dos principais fatores de risco e proteção para DCNT no Brasil^18^.

O processo amostral do Vigitel ocorreu em dois estágios. No primeiro, foi realizado sorteio das linhas telefônicas fixas e móveis por cidade. Já na segunda etapa, foi selecionado um adulto (≥18 anos de idade) para a entrevista, sorteado entre os residentes do domicílio. A amostragem incluiu 800 entrevistas por cidade, sendo 400 realizadas por telefone fixo e 400 por telefone móvel. Essa metodologia garantiu estimativas com um intervalo de confiança de 95% (IC95%) e margem de erro de até quatro pontos percentuais para a frequência de fatores de risco e proteção na população adulta de cada localidade. Informações detalhadas sobre o processo de amostragem do Vigitel podem ser encontradas no relatório publicado^18^.

### Variáveis do estudo

#### Variável desfecho – depressão

A presença de depressão foi definida por meio da pergunta “*Algum médico já lhe informou que o(a) senhor(a) tem depressão?*”, sendo categorizada como “sim” ou “não”.

#### Variável explicativa – coocorrência de comportamentos de risco à saúde

A coocorrência de comportamentos de risco à saúde foi avaliada com base na soma de cinco comportamentos: consumo de alimentos ultraprocessados (AUP), inatividade física, CS, tabagismo e consumo abusivo de álcool.

O consumo de AUP foi analisado com base em uma pergunta referente ao consumo de 13 subgrupos alimentares no dia anterior à entrevista: “*Agora vou listar alguns alimentos e gostaria que você me dissesse se comeu algum deles ontem (desde que acordou até quando foi dormir): bebidas adoçadas (refrigerantes, sucos prontos, refrescos em pó, bebidas achocolatadas), lanches industrializados (salgadinhos, biscoitos doces e salgados, bolinhos de pacote), sobremesas processadas (chocolates, sorvetes, gelatinas, flans), embutidos (salsicha, linguiça, mortadela, presunto), pães industrializados (pão de forma, pão de cachorro-quente, pão de hambúrguer), condimentos (maionese, ketchup, mostarda), margarinas e refeições prontas (macarrão instantâneo, sopas de pacote, pratos congelados)*”. Os subgrupos de AUP foram selecionados entre aqueles mais consumidos no Brasil, de acordo com a Pesquisa de Orçamentos Familiares (POF) de 2008–2009^19^. Para fins de análise, um escore foi calculado com base nas respostas positivas para o consumo de cada um dos subgrupos de AUP, variando entre 0 e 13 pontos. Com base na pontuação final, o consumo de AUP foi considerado alto quando igual ou superior a cinco subgrupos, sendo categorizado como “não” (<5 subgrupos) ou “sim” (≥5 subgrupos)^20^.

A inatividade física foi avaliada com base na classificação dos participantes como fisicamente inativos. Consideraram-se fisicamente inativos aqueles que, nos últimos três meses, não praticaram nenhuma atividade física (AF) no tempo livre, não realizaram esforços físicos intensos no trabalho, não se deslocaram a pé ou de bicicleta para o trabalho ou para a escola/cursos e não realizaram tarefas domésticas pesadas. Este indicador foi construído com base nas questões do Vigitel que avaliam os quatro domínios de AF: tempo livre, deslocamento, ocupacional e doméstico^18^.

O CS foi avaliado pelo tempo de tela, pelas perguntas: “*Em média, quantas horas por dia o(a) Sr.(a) costuma assistir à televisão?*” *e* “*Em média, quantas horas do seu tempo livre (excluindo o trabalho) são ocupadas pelo uso de computador, tablet ou celular?*”. Os participantes foram classificados “sem CS” ou “com CS”, sendo esta última categoria atribuída àqueles que referiram três ou mais horas diárias em CS^18^.

O tabagismo foi mensurado por meio da pergunta: “*Atualmente, o(a) Sr.(a) fuma?*”. Foi considerado fumante o indivíduo que respondeu positivamente, independentemente do número de cigarros, da frequência ou da duração do hábito de fumar.

Para avaliar o consumo abusivo de álcool, foram utilizadas as seguintes questões, direcionadas a homens e mulheres, respectivamente: “*Nos últimos 30 dias, o Sr. chegou a consumir 5 ou mais doses de bebida alcoólica em uma única ocasião?*” e “*Nos últimos 30 dias, a Sra. chegou a consumir 4 ou mais doses de bebida alcoólica em uma única ocasião?*”. Considerou-se uma dose de bebida alcoólica o equivalente a uma lata de cerveja, uma taça de vinho ou uma dose de cachaça, uísque ou qualquer outra bebida alcoólica destilada. O consumo abusivo foi definido como a ingestão de cinco ou mais doses, no caso dos homens, ou quatro ou mais doses, no caso das mulheres, em uma única ocasião em relação aos últimos 30 dias anteriores à data da pesquisa^18^.

Com base nas respostas aos cinco comportamentos, foi criada uma variável de coocorrência de comportamentos de risco à saúde, cuja pontuação variou de zero a cinco. Para fins de análise, essa variável foi categorizada em: “nenhum”, “um”, “dois” e “três ou mais” comportamentos de risco à saúde.

### Covariáveis

Foram avaliadas as seguintes características sociode-mográficas e antropométrica dos indivíduos: sexo (masculino/feminino); idade (18–24; 25–34; 35–44; 45–54; 55–64; ≥65 anos); escolaridade (0–8; 9–11; ≥12 anos de estudo); estado civil (com ou sem parceiro/cônjuge); e excesso de peso, definido como índice de massa corporal (IMC) ≥25 kg/m^2^, calculado de acordo com o peso e a altura autorrelatados^18^.

### Análise estatística

Foram realizadas análises descritivas por meio da distribuição de frequência (%) e respectivos IC95%. O teste de qui-quadrado de Pearson foi utilizado para avaliar a relação entre as covariáveis e a presença de depressão.

A associação entre cada comportamento de risco à saúde, de forma individual, e a presença de depressão foi avaliada por meio de regressão logística univariada. Posteriormente, foi realizada análise de regressão logística múltipla entre a variável explicativa e o desfecho, com estimativas brutas e ajustadas pelas covariáveis avaliadas neste estudo. A medida de associação utilizada foi a razão de chances (RC), com IC 95%. As variáveis de ajuste foram selecionadas com base em evidências da literatura sobre associação com a depressão e os comportamentos de risco à saúde^21,22,23^.

Para a análise dos dados, foi utilizado o *software Stata*, versão 18.0, no módulo *survey*, que considera os efeitos da amostragem complexa e permite a extrapolação dos resultados para a população brasileira. Foi adotado um nível de significância de 5% em todas as análises estatísticas.

### Aspectos éticos

O projeto Vigitel foi aprovado pela Comissão Nacional de Ética em Pesquisa para Seres Humanos do Ministério da Saúde (CAAE: 65610017.1.0000.0008). As bases de dados do Vigitel são de acesso público e podem ser consultadas no site oficial do Departamento de Análise Epidemiológica e Vigilância de Doenças Não Transmissíveis (DAENT): https://svs.aids.gov.br/download/Vigitel/.

#### Declaração de disponibilidade de dados

Os dados do Vigitel estão disponíveis para acesso público no seguinte URL: https://svs.aids.gov.br/daent/cgdnt/vigitel/

## RESULTADOS

Foram avaliados 21.690 indivíduos, sendo a maioria do sexo feminino, com idade entre 25 e 54 anos, escolaridade entre 9 e 11 anos, sem companheiro e com excesso de peso. O diagnóstico médico de depressão foi relatado por 12,3%. Com exceção da variável idade, todas as demais covariáveis estiveram relacionadas com a depressão (Tabela 1).

**Tabela 1:** Características sociodemográficas e antropométricas segundo a presença de depressão entre adultos e idosos brasileiros (n=21.690). Vigitel, 2023.

Variáveis	Frequência (%, IC95%)	Depressão (%, IC95%)		Valor-p
		Ausência	Presença	
		87,7 (86,8-88,5)	12,3 (11,5-13,2)	
Sexo				
Masculino	46,0 (44,5–47,6)	92,9 (91,8–93,9)	7,1 (6,1-8,2)	**<0,001**
Feminino	54,0 (52,4–55,5)	83,2 (81,9–84,5)	16,8 (15,5-18,2)	
Idade (anos)				
18 a 24	12,9 (11,8–14,0)	89,2 (86,1–91,6)	10,8 (8,4-13,9)	0,152
25 a 34	24,9 (23,4–26,4)	88,9 (86,7–90,7)	11,1 (9,3-13,3)	
35 a 44	18,3 (17,2–19,4)	86,5 (84,3–88,4)	13,5 (11,6-15,7)	
45 a 54	18,3 (17,2–19,5)	88,2 (86,2–90,0)	11,8 (10,0-13,8)	
55 a 64	13,9 (12,9–14,8)	86,6 (84,5–88,5)	13,4 (11,6-15,6)	
≥ 65	11,7 (11,1–12,5)	85,7 (84,0–87,4)	14,3 (12,6-16,1)	
Escolaridade (anos)				
0 a 8	32,9 (31,6–34,3)	86,0 (84,3–87,5)	14,0 (12,5-15,7)	**0,016**
9 a 11	41,3 (39,8–42,8)	89,0 (87,6–90,2)	11,0 (9,9-12,4)	
≥12	25,8 (24,4–27,2)	87,8 (85,9–89,4)	12,2 (10,6-14,1)	
Estado civil				
Com companheiro	46,5 (45,0–48,0)	89,1 (88,0–90,2)	10,9 (9,7-12,1)	**0,001**
Sem companheiro	53,5 (52,0–55,0)	86,3 (84,9–87,5)	13,7 (12,5-15,1)	
Excesso de peso				
Não	38,6 (37,2–40,1)	89,0 (87,7–90,2)	11,0 (9,8-12,3)	**0,015**
Sim	61,4 (59,9–62,8)	86,8 (85,6–88,0)	13,2 (12,0-14,4)	

Nota: Depressão: avaliada com base no autorrelato.

IMC: Índice de Massa Corporal ≥25 kg/m^2^(excesso de peso).

Valor de p obtido por meio do teste de χ^2^de Pearson.

Em negrito, as variáveis que apresentaram significância estatística.

Entre os comportamentos avaliados, o CS foi o mais prevalente, correspondendo a 67,04% dos participantes (Tabela 2).

**Tabela 2:** Associação entre comportamentos de risco à saúde e depressão entre adultos e idosos do Brasil (n=21.690). Vigitel, 2023.

Comportamentos de risco à saúde	Prevalência (%) (%)	Depressão			
		Análise bruta OR (IC95%)	Valor-p	Análise ajustada OR (IC95%)	Valor-p
Consumo de alimentos ultraprocessados					
Não	82,25	1		1	
Sim	17,75	1,12 (0,91–1,38)	0,284	1,35 (1,08–1,68)	**0,008**
Inatividade física					
Não	86,12	1		1	
Sim	13,88	1,29 (1,04–1,60)	**0,021**	1,30 (1,05–1,62)	**0,018**
Comportamento sedentário					
Não	32,96	1		1	
Sim	67,04	1,11 (0,94–1,31)	0,220	1,11 (0,93–1,32)	0,233
Tabagismo					
Não	90,73	1		1	
Sim	9,27	1,76 (1,36–2,27)	**<0,001**	2,09 (1,60–2,73)	**<0,001**
Consumo abusivo de álcool					
Não	79,24	1		1	
Sim	20,76	0,99 (0,80–1,23)	0,921	1,18 (0,95–1,47)	0,131

Nota: Valores em negrito indicam significância estatística (p<0,05).

Regressão logística múltipla ajustada por sexo, idade, estado civil e escolaridade.

Consumo de alimentos ultraprocessados: categorizado como “não” (<5 subgrupos) e “sim” (≥5 subgrupos).

Inatividade física: classificada como “não” ou “sim”. Foi considerado fisicamente inativo o participante que não realizou qualquer atividade física nos últimos três meses em nenhum dos quatro domínios avaliados: lazer, deslocamento, ocupacional e doméstico.

Comportamento sedentário: categorizado como “não” para indivíduos sem comportamento sedentário e “sim” para aqueles que referiram dedicar três ou mais horas diárias a comportamentos sedentários.

Tabagismo: categorizado como “não” e “sim”. Considerou-se fumante o indivíduo que declarou fumar, independentemente do número de cigarros, da frequência ou da duração do hábito.

Consumo abusivo de álcool: classificado como “não” ou “sim”, sendo considerado “sim” quando houve ingestão de cinco ou mais doses, no caso dos homens, ou quatro ou mais doses, no caso das mulheres, em uma única ocasião.

Observou-se que o consumo de AUP, a inatividade física e o tabagismo estão associados à presença de depressão. Participantes com alto consumo de AUP têm mais chance de depressão (RC 1,35; IC95% 1,08– 1,68; p=0,008) em comparação àqueles com consumo de AUP<5 subgrupos. Indivíduos fisicamente inativos apresentaram maior chance de depressão (RC 1,29; IC95% 1,04–1,60; p=0,021) quando comparados aos indivíduos ativos. Além disso, participantes fumantes tiveram maior chance de apresentar depressão (RC 1,76; IC95% 1,36–2,27; p<0,001), em comparação com os não fumantes (Tabela 2).

A análise de regressão múltipla entre a coocorrência de comportamentos de risco à saúde e depressão mostrou um gradiente dose-resposta (p<0,001), indicando que a chance de apresentar depressão aumenta quanto maior é o número de comportamentos de risco à saúde. A presença de apenas um comportamento não esteve associada à depressão. Participantes com dois comportamentos apresentaram 1,36 vezes mais chance de depressão (RC 1,36; IC95% 1,08–1,73; p<0,010), enquanto aqueles com três ou mais comportamentos tiveram uma chance maior (RC 2,01; IC95% 1,49–2,71; p<0,001), em comparação aos que não apresentavam nenhum comportamento de risco à saúde (Tabela 3).

**Tabela 3:** Associação entre a coocorrência de comportamentos de risco à saúde e depressão entre adultos e idosos do Brasil (n=21.690). Vigitel, 2023.

Número de comportamentos de risco à saúde	Prevalência (%, IC95%)	Depressão		Valor-p
		Análise bruta (OR)	Análise ajustada (OR)	
Nenhum	20,17 (19,04–21,36)	-	-	<0,001^*^
Um	43,21 (41,73–44,71)	0,98 (0,80–1,20)	1,02 (0,83–1,26)	0,839
Dois	26,26 (24,96–27,60)	1,21 (0,97–1,53)	1,36 (1,08–1,73)	**0,010**
Três ou mais	10,35 (9,38–11,41)	1,50 (1,12–2,00)	2,01 (1,49–2,71)	**<0,001**

Nota: *p-tendência referindo-se ao teste de tendência linear.

Valores em negrito indicam significância estatística (p<0,05).

Comportamentos de risco à saúde: consumo de alimentos ultraprocessados, inatividade física, comportamento sedentário, tabagismo e consumo abusivo de álcool.

Regressão logística múltipla ajustada por sexo, idade, estado civil e escolaridade.

## DISCUSSÃO

Os resultados do presente estudo, que incluiu mais de 21 mil adultos e idosos brasileiros, revelaram uma prevalência de 12,3% de diagnóstico médico de depressão e que 36,61% dos participantes apresentaram dois ou mais comportamentos de risco à saúde. A análise individual dos comportamentos indicou que o consumo de AUP, a inatividade física e o tabagismo estiveram associados à depressão. Além disso, observou-se uma associação estatisticamente significativa entre a coocorrência dos comportamentos de risco à saúde e a presença de depressão, evidenciando que o aumento do número de comportamentos esteve relacionado a uma maior chance de diagnóstico depressivo.

De acordo com dados do relatório *The Burden of Mental Disorders in the Region of the Americas*, a depressão é o transtorno mental mais incapacitante nas Américas, sendo responsável por cerca de 7,8% dos anos vividos com incapacidade (*Years Lived with Disability* – YLDs)^6^. No Brasil, o cenário torna-se particularmente preocupante à medida que pesquisas nacionais de base populacional, como Vigitel e PNS, evidenciam uma tendência crescente e contínua nas taxas de depressão ao longo das últimas décadas^7,19^.

O aumento da prevalência da depressão é particularmente preocupante quando associada ao estilo de vida da população, especialmente aqueles relacionados aos comportamentos de risco à saúde. Evidências indicam uma relação bidirecional entre a depressão e o consumo alimentar inadequado, CS e outros comportamentos, configurando um ciclo vicioso que compromete tanto a saúde física quanto a mental^9,24,25^.

No presente estudo, o consumo de AUP esteve associado à presença de depressão, corroborando os achados de Hecht et al.^26^. Esses autores observaram que indivíduos com maior consumo de AUP apresentaram maior probabilidade de relatar depressão leve, maior número de dias com sofrimento mental e sintomas de ansiedade, bem como menor probabilidade de relatar ausência de comprometimento da saúde mental.

A associação entre o consumo frequente de AUP e aumento nos níveis de sintomas depressivos^27^ pode ser explicada pelos efeitos adversos desses alimentos sobre a saúde metabólica e o funcionamento neuropsicológico^28^. Estudos indicam que o consumo de AUP pode desencadear inflamação sistêmica por meio da ativação de vias pró-inflamatórias e do aumento da permeabilidade da barreira intestinal, favorecendo o desenvolvimento de disbiose intestinal. Esse processo inflamatório apresenta potencial para comprometer a neurotransmissão, especialmente nos sistemas serotoninérgico e dopaminérgico, que desempenham papel fundamental na regulação do humor^29,30^.

No presente estudo, a inatividade física esteve associada a maior chance de depressão, em consonância com evidências que apontam que comportamentos de movimento inadequados estão relacionados a transtornos mentais^15,16^. Há evidências de que esses comportamentos influenciam a saúde mental por meio de alterações na função cerebral e na regulação do humor^31,32^. Além disso, diferentes níveis de intensidades de AF podem exercer efeitos protetores sobre a depressão, atuando por meio de mecanismos biológicos e psicossociais^16,33^.

A inatividade física é associada à redução na liberação de neurotransmissores essenciais para a regulação do humor e do bem-estar, como dopamina, serotonina e endorfina^31,34^. Além disso, a ausência de AF compromete processos neurobiológicos importantes, como a neurogênese e a plasticidade cerebral^33,34^. Indivíduos fisicamente inativos são mais propensos ao acúmulo de estresse psicológico, uma vez que a AF desempenha um papel fundamental na regulação dos sistemas de resposta ao estresse, como o eixo Hipotálamo-Hipófise-Adrenal (HPA)^31,35^, sistema neuroendócrino responsável pela resposta fisiológica adequada diante de situações estressoras^33^.

Diversos mecanismos têm sido propostos para explicar a associação entre CS e depressão. Uma das hipóteses é que níveis elevados de CS podem exercer efeitos deletérios diretos sobre o humor, independentemente de outros fatores, possivelmente por meio de alterações nos processos inflamatórios, os quais constituem uma das principais características biológicas da depressão maior^16^. O CS também está associado ao aumento da inflamação sistêmica, evidenciado por níveis elevados de marcadores inflamatórios, como proteína C-reativa e interleucina-6, que podem afetar o funcionamento do sistema nervoso central, favorecendo alterações neuroquímicas e estruturais que elevam o risco de desenvolvimento de depressão^16,35,36^.

Outra hipótese sugere que CS baseado em telas, como o uso prolongado de computadores, pode contribuir para a redução das interações sociais presenciais, o que, por sua vez, estaria associado ao aumento do risco de desenvolvimento de transtornos mentais^16,36^. Por fim, longos períodos gastos em CS podem resultar na redução da prática de AF, o que leva a uma diminuição da liberação de neurotransmissores associados à regulação positiva do humor, como as endorfinas — contribuindo, assim, para sintomas depressivos^36^.

Assim como os comportamentos de movimento, o tabagismo também esteve associado à presença de depressão neste estudo, corroborando com Völker et al.^37^. Em estudo com mais de 170 mil participantes, os autores observaram que indivíduos que haviam fumado anteriormente apresentavam maiores chances de depressão ao longo da vida e no momento atual, além de associação entre maior consumo de cigarros e maior gravidade de sintomas depressivos^37^.

O uso de tabaco tem sido identificado como um importante comportamento de risco para a depressão, com efeitos possivelmente mediados por mecanismos neurobiológicos e inflamatórios. Evidências apontam que indivíduos fumantes apresentam níveis mais elevados de depressão em comparação aos não fumantes^27,37^, sugerindo que o hábito de fumar pode afetar negativamente a neuroquímica cerebral, favorecendo o desenvolvimento de quadros depressivos^38^. A nicotina, principal substância psicoativa do tabaco, estimula a liberação de neurotransmissores no sistema de recompensa cerebral, gerando efeitos prazerosos^39^. Contudo, o seu uso crônico pode levar à desregulação desses neurotransmissores, contribuindo para o desenvolvimento da depressão^40^. Além disso, o tabagismo está associado a um estado inflamatório sistêmico, caracterizado pelo aumento de citocinas pró-inflamatórias, que podem comprometer a neurogênese e afetar regiões cerebrais envolvidas na regulação do humor^41^.

Neste estudo, a coocorrência de comportamentos de risco à saúde esteve associada à depressão. Observou-se que, quanto maior o número de comportamentos de risco, maior a chance de depressão, corroborando os achados de Barbosa et al.^9^ em um estudo transversal realizado com 1.353 jovens adultos que avaliou a coocorrência de comportamentos obesogênicos e a presença de sintomas de ansiedade e depressão. Além disso, em estudo longitudinal, Kekäläinen et al.^42^ encontraram associações acumuladas entre comportamentos de risco à saúde e bem-estar mental ao longo de 30 anos, desde o início da vida adulta até fases mais tardias. A presença de múltiplos comportamentos, como tabagismo, consumo excessivo de álcool e inatividade física, esteve associada a pior bem-estar mental.

Evidências indicam que comportamentos de risco à saúde frequentemente tendem a ocorrer de forma agrupada, e que sua coocorrência pode exercer efeito sinérgico, potencializando a influência de um comportamento sobre os demais^9,13,21^. Além disso, estudos epidemiológicos demonstram que essa combinação está associada a desfechos em saúde mental^9,42^.

Diferentes mecanismos podem explicar a associação entre comportamentos de risco à saúde e transtornos mentais. Alterações nos hábitos alimentares, por exemplo, podem ser reflexo do comer emocional ou da falta de motivação para manter padrões alimentares saudáveis. Nesse contexto, indivíduos com maior percepção de estresse e sintomas depressivos tendem a aumentar o consumo de AUP como forma de lidar com as emoções^9^. Ademais, esses alimentos têm sido associados ao aumento dos sintomas depressivos, possivelmente em decorrência de seus efeitos negativos sobre a saúde metabólica e neuropsicológica. Da mesma forma, a literatura sugere que fumar pode influenciar a neuroquímica cerebral e os padrões de inflamação, contribuindo para a depressão^27^.

Embora os resultados deste estudo sejam de grande relevância, é fundamental considerar algumas limitações. O delineamento transversal do estudo não permite identificar temporalidade nem estabelecer relação de causa-efeito. É importante ressaltar a possibilidade de causalidade reversa, particularmente no que se refere à depressão e aos comportamentos de risco, incluindo tabagismo, hábitos alimentares inadequados e inatividade física. Além disso, a composição da amostra, restrita a indivíduos com acesso a linhas telefônicas, pode comprometer a representatividade dos dados em relação à população geral, especialmente em populações mais vulneráveis. A natureza autorreferida das informações também apresenta desafios metodológicos, expondo os dados a potenciais vieses de memória e interpretação. Para atenuar essas limitações, foram aplicados fatores de ponderação que ajustam a composição sociodemográfica da amostra, aumentando tanto a representatividade quanto a robustez analítica do estudo. Por fim, deve-se destacar que a avaliação dos comportamentos de risco de forma combinada limita a exploração de possíveis interações e padrões mais complexos, diferentemente de abordagens como a análise de *clusters*.

Mesmo diante dessas limitações, este estudo apresenta contribuições relevantes ao elucidar a associação entre comportamentos de risco à saúde e depressão na população brasileira. A metodologia aplicada possibilitou não apenas identificar as associações, mas também destacar a interdependência entre diversos comportamentos prejudiciais à saúde e seu impacto na depressão. Essa abordagem holística enfatiza a importância de estratégias de saúde que integrem, simultaneamente, ações voltadas para a saúde mental e a promoção de hábitos de vida saudáveis.

A originalidade deste artigo está na análise combinada de múltiplos comportamentos de risco à saúde e sua interação com a saúde mental, oferecendo uma perspectiva mais abrangente e detalhada sobre duas questões importantes de saúde pública: DCNT e saúde mental. Os achados deste estudo reforçam a necessidade de políticas públicas abrangentes, capazes de articular diferentes estratégias e promover uma visão integrada sobre os determinantes da saúde. Intervenções que estimulem a prática regular de AF e incentivem padrões alimentares adequados e saudáveis, aliadas à redução simultânea dos comportamentos de risco à saúde, podem desempenhar papel central na prevenção e no controle das DCNT e dos transtornos mentais. A incorporação dessas ações ao cotidiano da população representa uma oportunidade de promover a saúde de forma mais ampla e sustentável.

## APROVAÇÃO CEP

Os dados utilizados neste estudo são de domínio público, provenientes do inquérito telefônico de base populacional Vigitel 2023. A aprovação ética do Vigitel foi obtida pelo Comitê Nacional de Ética em Pesquisa com Seres Humanos do Ministério da Saúde (CAAE: 65610017.1.0000.0008).
